# Supplementary material for: MEOX2 Transcription Factor Is Involved in Survival and Adhesion of Glioma Stem-like Cells
Source: Cancers (Basel). 2021 Nov 25;13(23):5943. doi: 10.3390/cancers13235943 (PMC8672280; doi:10.3390/cancers13235943)

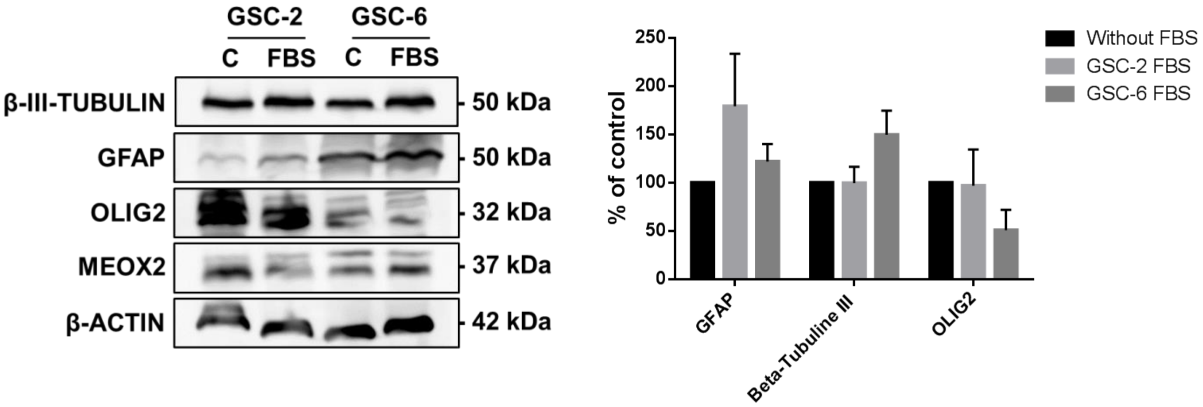

Figure S1. Representative WB and histograms of markers of differentiation in GSC-2 and GSC-6 after 6 days of culture in 5% Fetal Bovine Serum (FBS).

	GSC-2	GSC -6	GSC -10	GSC -11	GSC -7	GSC -5
**Patient/Tumor**						
**Age**	57	53	69	62	64	65
**Gender**	M	M	M	M	M	F
**Resection**	Yes	Yes	Yes	Yes	ND	Yes
**biopsy**	No	No	No	No	ND	No
**Radiotherapy**	Yes	Yes	Yes	Yes	Yes	Yes
**Chemotherapy (Temozolomid)**	Yes	Yes	Yes	Yes	Yes	Yes
**Progression free survival (months)**	3	2	10	10	43 (censored)	6
**Overall survival (months)**	9	4	25	27	43 (censored)	11
**Glioma Stem Cell**						
**Chromosomal traits**						
**Codel 1p19q**	Negative	Negative	Negative	Negative	Negative	Negative
**Gain 7**	Positive	Positive	Negative	Positive	Positive	Positive
**Ampli EGFR**	Negative	Negative	Negative	Negative	Negative	Negative
**Perte 9p (+/- del suppl CDKN2A/B)**	Positive	*CDKN2A/B*	Negative	Positive	Negative	Positive
**chr10 (positive si PTEN inclus)**	Positive	Positive	Positive	Positive	Positive	Negative
**Gain 12 ou Amp MDM2 ou CDK4**	Positive	Negative	Negative	Negative	Negative	Positive
**Molecular traits**						
***IDH1* status**	Wild Type	Wild Type	Wild Type	Wild Type	Wild Type	Wild Type
***IDH2* status**	Wild Type	Wild Type	Wild Type	Wild Type	Wild Type	Wild Type
***TP53* status**	Wild Type	Mutated	Wild Type	Wild Type	NC	Mutated
***PTEN* status**	Mutated	Mutated	Mutated	Wild Type	NC	Wild Type
**EGFR vIII mutation**	Wild Type	Mutated	Mutated	Mutated	Wild Type	Mutated
***MGMT* methylation**	1.6%	34.2%	86.8%	94.2%	19.2%	2%
**BRAF ^{V600E} status**	Wild Type	Wild Type	Wild Type	Mutated	Wild Type	Wild Type
**NGS**	Wild Type	Wild Type	Wild Type	Wild Type	Wild Type	Wild Type
**Other traits**						
**Morphology**	Sphere	Adherent	Sphere	Semi-sphere	Sphere	Sphere
**Verhaak classification**	Proneural	Mesenchymal	Classique	Mesenchymal	Mesenchymal	Mesenchymal

Table S1: Patient, tumor and GSC features.

Patients	Date of birth	Date at diagnosis	Age	Grade	IDH status	1p19q codeletion	MGMT methylation statut (%)	MEOX2 methylation statut (%)
1	11/17/1976	7/23/2019	43	grade III	R132H	Codeletion	38	63.7
2	2/2/1977	8/14/2019	43	grade III	R132H	Codeletion	23	42.3
3	10/17/1978	11/12/2013	35	grade III	R132H	Codeletion	51	60.3
4	4/4/1976	2/11/2020	44	grade III	R132H	Codeletion	59	66.7
5	1/24/1979	2/13/2020	41	grade II	R132H	Codeletion	29	51.7
6	3/23/1952	4/8/2019	67	grade III	R132H	Codeletion	24	83,0
7	7/28/1960	4/19/2019	59	grade III	R132H	Codeletion	53	92,0
8	8/7/1979	2/4/2019	40	grade II	R132H	Codeletion	44	54.3
9	1/14/1971	6/25/2019	49	grade II	R132H	Codeletion	40	79,0
10	2/24/1995	11/6/2019	25	grade II	R132H	Codeletion	15	32,0
11	10/12/1963	12/27/2019	56	grade III	R132H	Codeletion	47	97.0
12	8/19/1970	9/9/2019	49	grade II	R132H	WT	42	13.7
13	3/24/1981	10/9/2019	39	grade III	R132H	WT	42	66.3
14	6/9/1956	3/24/2020	64	grade II	R132H	WT	40	82.0
15	2/17/1977	12/16/2019	43	grade II	R132H	WT	13	84.0
16	2/16/1965	8/28/2019	55	grade III	R132H	WT	33	63.3
17	1/26/1987	4/18/2019	32	grade III	R132G	WT	24	74.0
18	3/25/1994	6/17/2019	25	grade II	R132H	WT	19	54.3
19	9/27/1983	5/21/2019	36	grade II	R132H	WT	37	65.0
20	10/5/1961	4/2/2019	58	grade III	R132H	WT	28	63.7
21	1/18/1992	2/27/2020	28	grade III	R132H	WT	NC	60.0
22	9/8/1977	6/25/2019	42	grade IV	R132H	WT	38	62,0
23	12/12/1968	2/20/2019	50	grade IV	R132H	WT	70	76.7
24	5/25/1979	1/31/2020	41	grade IV	R132H	WT	28	72.7
25	10/18/1951	3/13/2020	69	grade IV	WT	WT	4	9.0
26	4/11/1963	3/19/2020	57	grade IV	WT	WT	2	5.0
27	12/27/1949	3/18/2020	70	grade III	WT	WT	4	9.3
28	10/31/1951	3/23/2020	69	grade IV	WT	WT	23	5.7
29	8/8/1957	10/3/2019	62	grade IV	WT	WT	5	3.7
30	7/31/1945	7/16/2019	74	grade III	WT	WT	2	2.7
31	6/28/1948	9/10/2019	71	grade IV	WT	WT	32	12.0
32	3/10/1951	11/26/2019	69	grade IV	WT	WT	3	2.3
33	4/23/1940	9/11/2019	80	grade IV	WT	WT	4	3.3
34	12/17/1954	3/23/2020	65	grade IV	WT	WT	3	4.7
35	1/15/1959	3/16/2020	61	grade IV	WT	WT	12	6.0
36	8/22/1978	3/23/2020	42	grade IV	WT	WT	21	12.3
37	4/2/1957	3/12/2020	63	grade IV	WT	WT	6	5.3

Table S2: Molecular feature of patient cohort.

Name	Species	Reference	RRID	Supplier	WB Dilution	IF Dilution
ß-ACTIN	Mouse	A5316	AB_476743	Sigma-Aldrich	1:5000	
DCX	Goat	sc-8066	AB_2088494	Santa Cruz Biotechnology	1:200	
GAPDH	Mouse	MAB374	AB_2107445	Merck Millipore	1:2000	
LAMIN A / C	Mouse	4777	AB_10545756	CellSignaling Technology	1:1000	
GFAP	Mouse	G3893	AB_477010	Sigma-Aldrich	1 :500	
OLIG2	Rabbit	AB9610	AB_570666	Merck Millipore	1:250	
AKT	Rabbit	9272	AB_329827	CellSignaling Technology	1:500	
pT308-AKT	Rabbit	13038	AB_2629447	CellSignaling Technology	1:350	
MEOX2	Rabbit	HPA053793	AB_2682264	Sigma-Aldrich	1:500	1:500
CDH10	Rabbit	PAB3147	AB_1571934	Abnova	1:500	1:250
ERK (44 / 42)	Rabbit	9102	AB_330744	CellSignaling Technology	1:500	
pT202/Y204-ERK (44 / 42)	Rabbit	9101	AB_331646	CellSignaling Technology	1:500	
MAP-2	Mouse	MAB-3418	AB_94856	Merck Millipore	1:500	
FAK	Rabbit	71433S	AB_2799801	CellSignaling Technology	1:500	
pY397-FAK	Rabbit	8556S	AB_10891442	CellSignaling Technology	1:500	
PARP	Rabbit	9542	AB_2160739	CellSignaling Technology	1 :500	
Phalloidine Alexa fluor 546		A22283	AB_2632953	Invitrogen		1 :700

Table S3: Antibodies.

Figure 1(a)

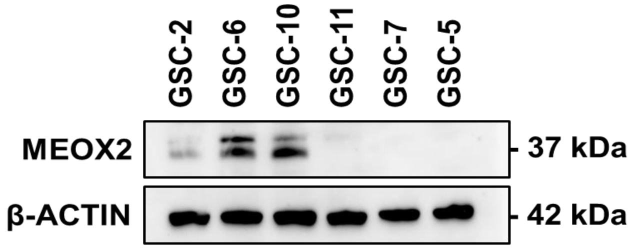
GSC-2
GSC-6
GSC-10
GSC-11
GSC-7
GSC-5
MEOX2
β-ACTIN
37 kDa
42 kDa

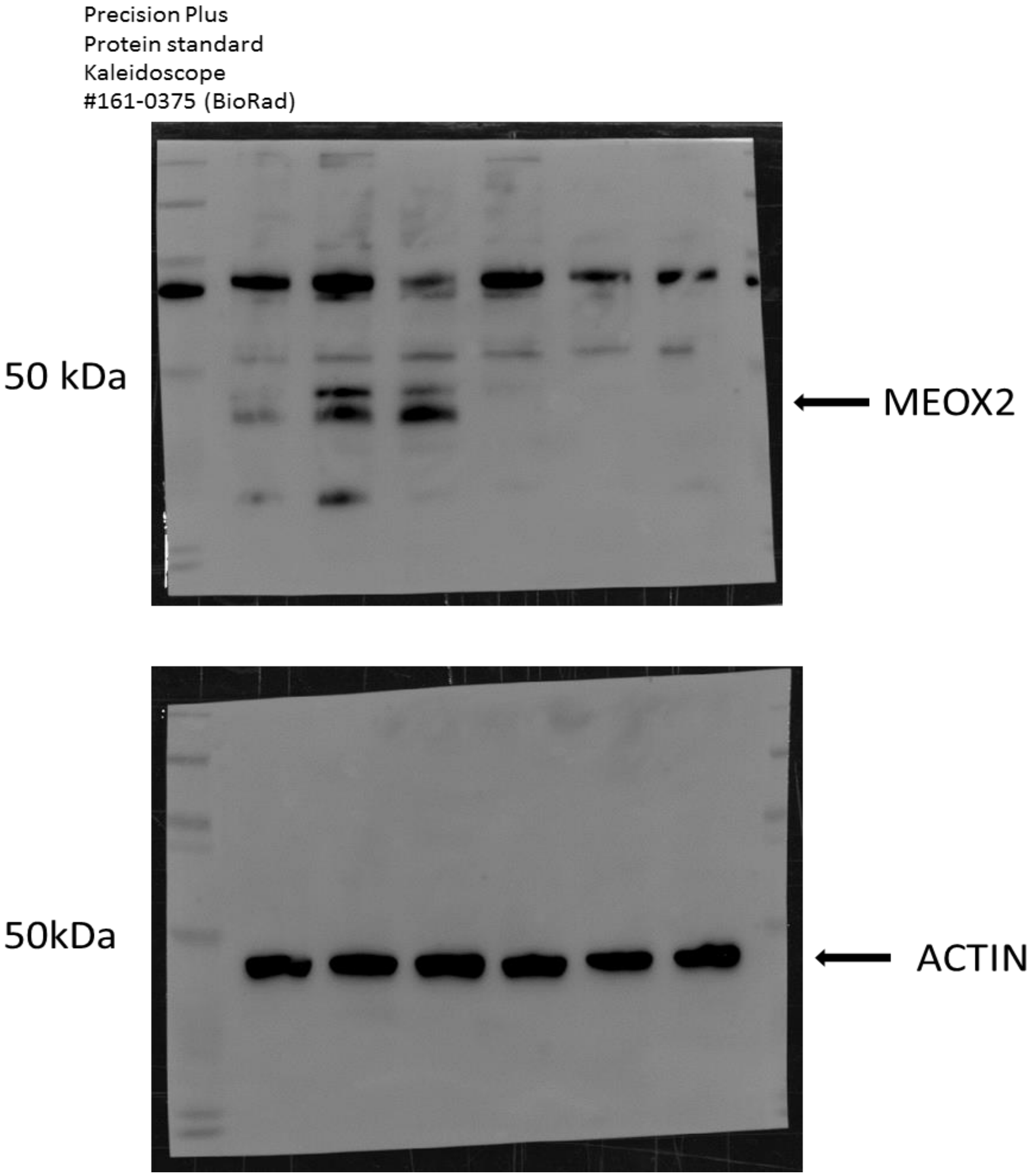
Precision Plus
Protein standard
Kaleidoscope
#161-0375 (BioRad)
50 kDa
MEOX2
50kDa
ACTIN

Figure 1(c)

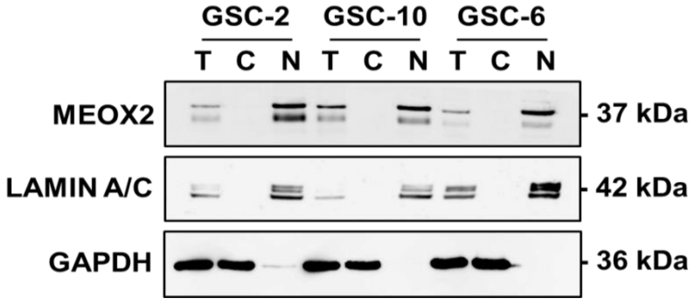
GSC-2
GSC-10
GSC-6
T C N T C N T C N
MEOX2
LAMIN A/C
GAPDH
37 kDa
42 kDa
36 kDa

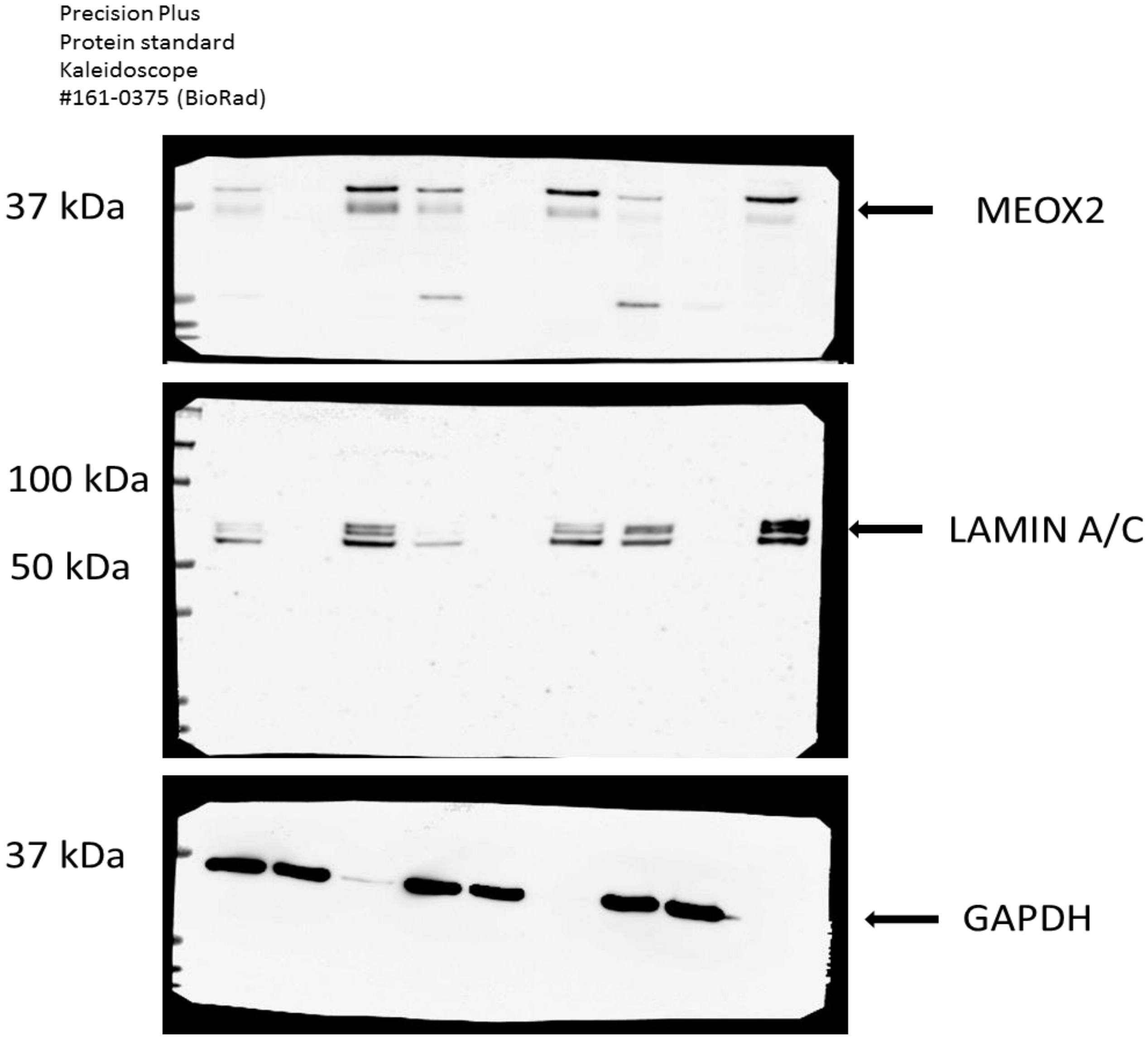
Precision Plus
Protein standard
Kaleidoscope
#161-0375 (BioRad)
37 kDa
MEOX2
100 kDa
50 kDa
LAMIN A/C
37 kDa
GAPDH

Figure 3(a)

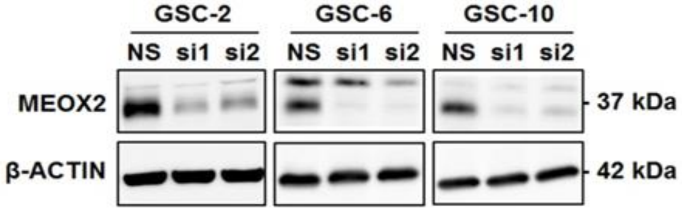
GSC-2
GSC-6
GSC-10
NS si1 si2
NS si1 si2
NS si1 si2
MEOX2
β-ACTIN
37 kDa
42 kDa

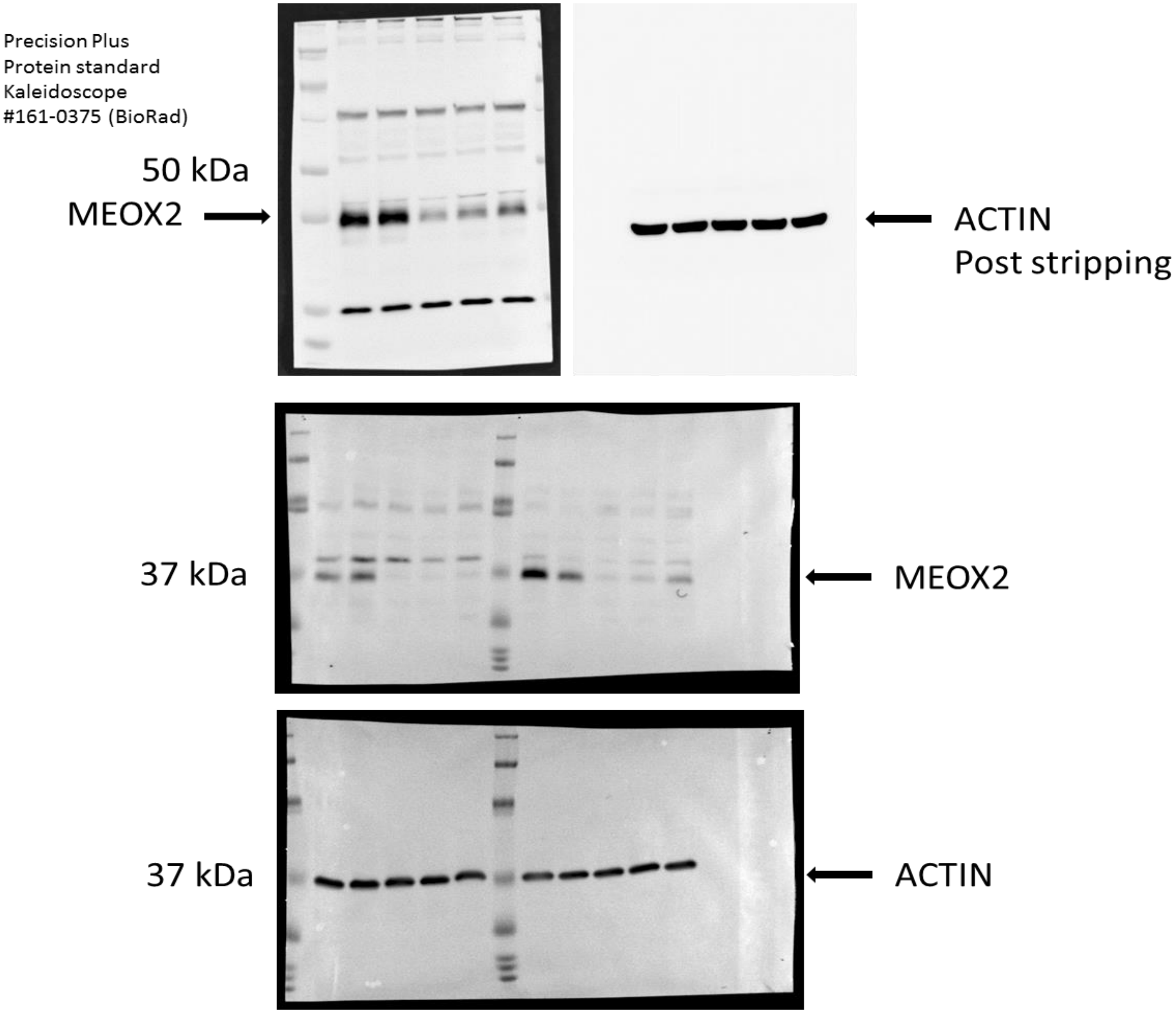
Precision Plus
Protein standard
Kaleidoscope
#161-0375 (BioRad)
50 kDa
MEOX2
ACTIN
Post stripping
37 kDa
MEOX2
37 kDa
ACTIN

Figure 3(c)

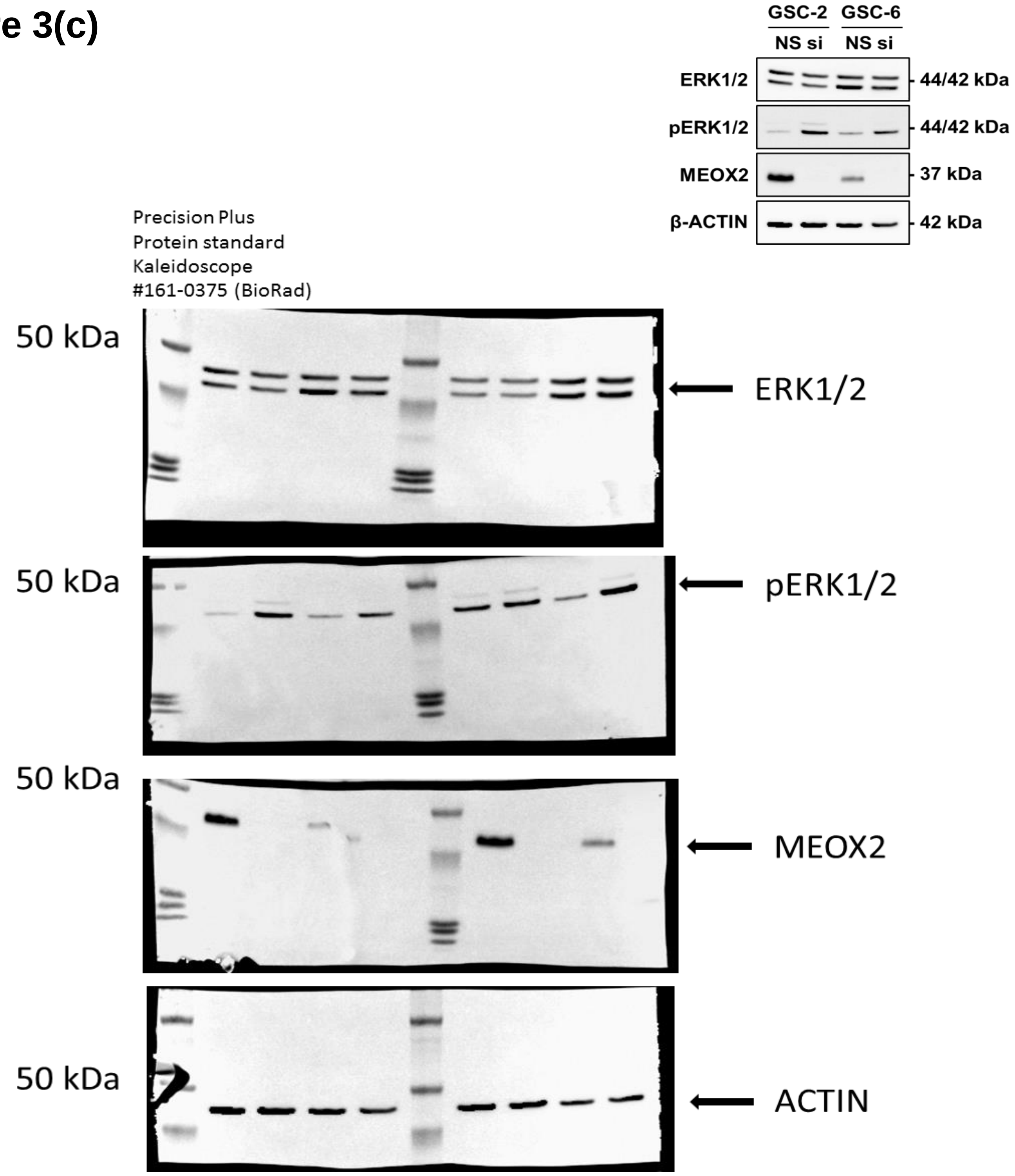
GSC-2
GSC-6
NS si
NS si
ERK1/2
pERK1/2
MEOX2
β-ACTIN
44/42 kDa
44/42 kDa
37 kDa
42 kDa
Precision Plus
Protein standard
Kaleidoscope
#161-0375 (BioRad)
50 kDa
ERK1/2
50 kDa
pERK1/2
50 kDa
MEOX2
50 kDa
ACTIN

Figure 4(b)

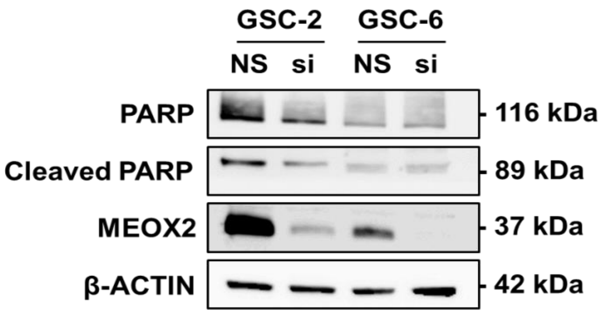

GSC-2
GSC-6
NS
si
NS
si
PARP
Cleaved PARP
MEOX2
β-ACTIN
116 kDa
89 kDa
37 kDa
42 kDa

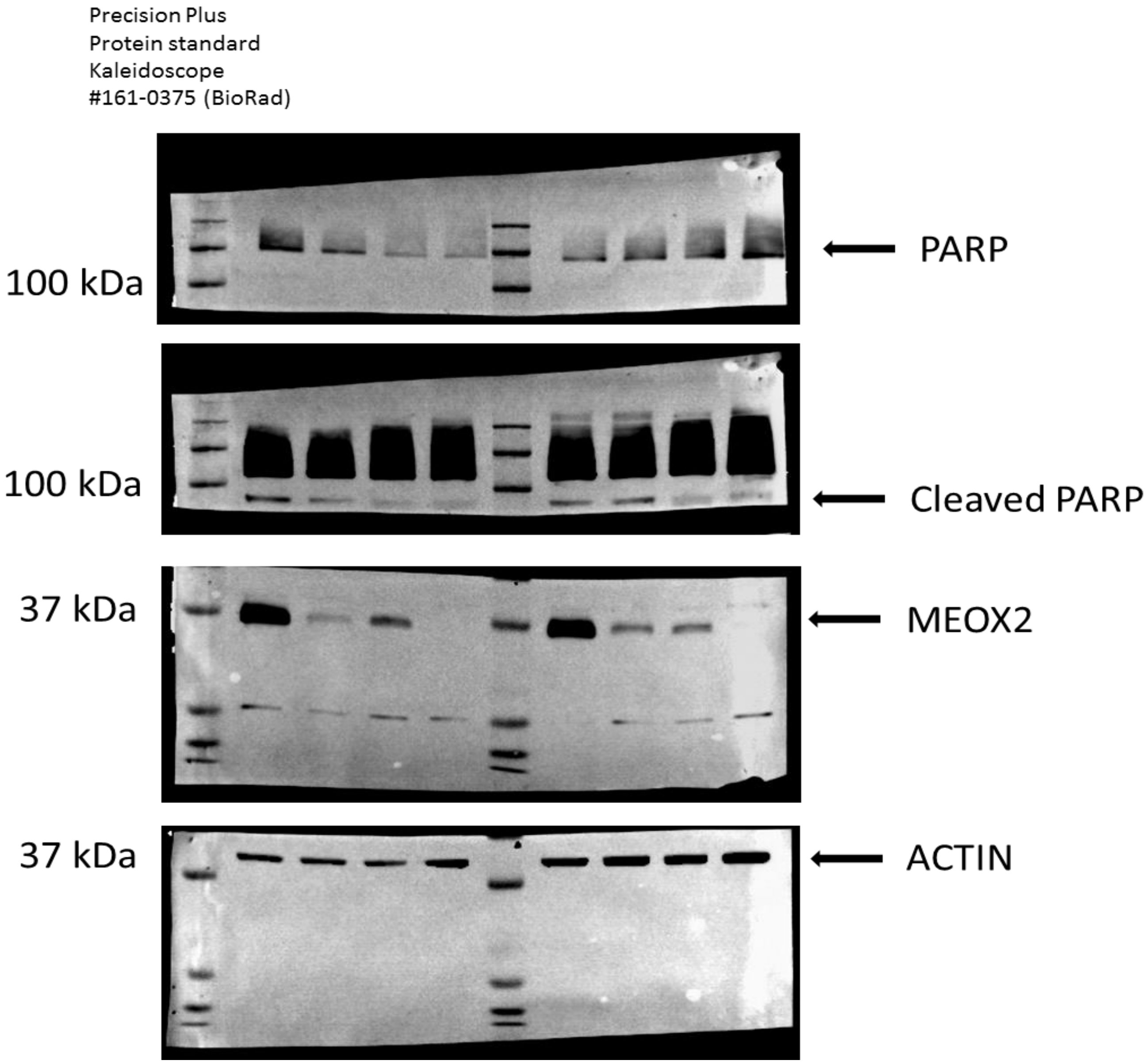

Precision Plus
Protein standard
Kaleidoscope
#161-0375 (BioRad)
100 kDa
PARP
100 kDa
Cleaved PARP
37 kDa
MEOX2
37 kDa
ACTIN

Figure 4(c)

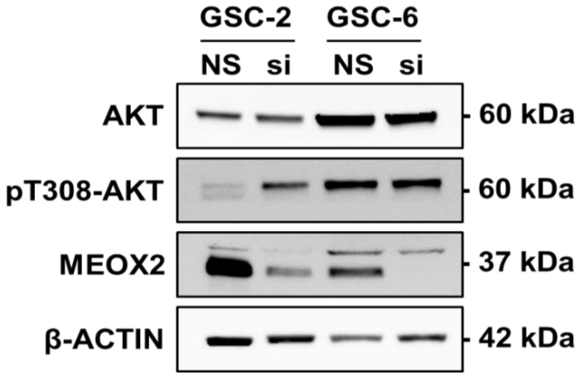
GSC-2
GSC-6
NS
si
NS
si
AKT
pT308-AKT
MEOX2
β-ACTIN
60 kDa
60 kDa
37 kDa
42 kDa

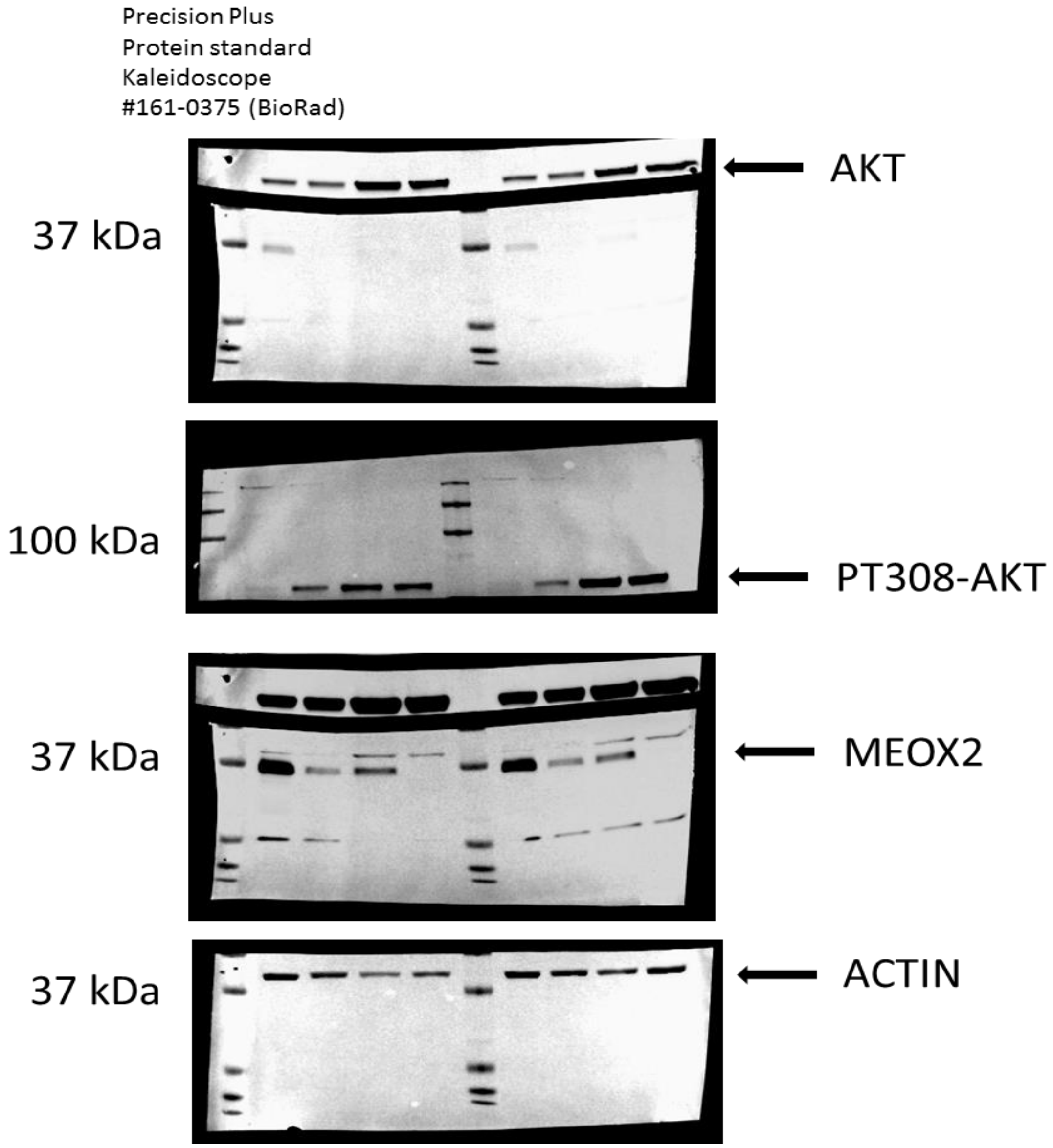
Precision Plus
Protein standard
Kaleidoscope
#161-0375 (BioRad)
37 kDa
AKT
100 kDa
PT308-AKT
37 kDa
MEOX2
37 kDa
ACTIN

Figure 5(a)

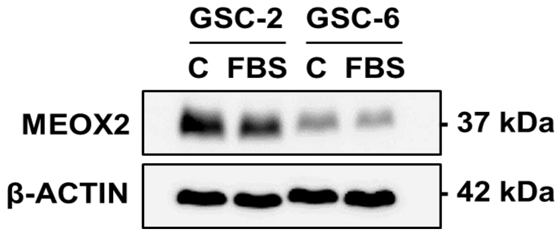

GSC-2
GSC-6
C
FBS
C
FBS
MEOX2
β-ACTIN
37 kDa
42 kDa

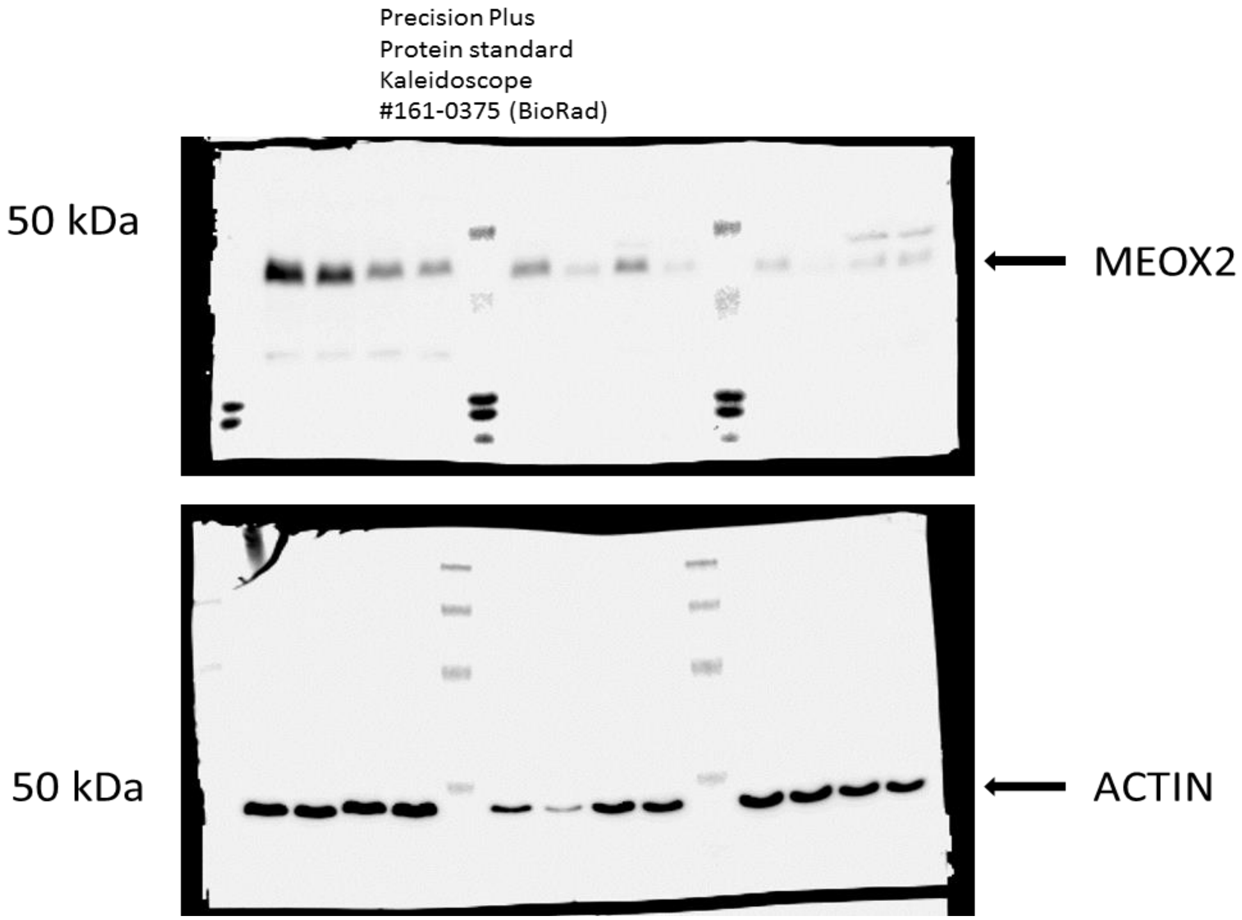

Precision Plus
Protein standard
Kaleidoscope
#161-0375 (BioRad)
50 kDa
MEOX2
50 kDa
ACTIN

Figure 5(c)

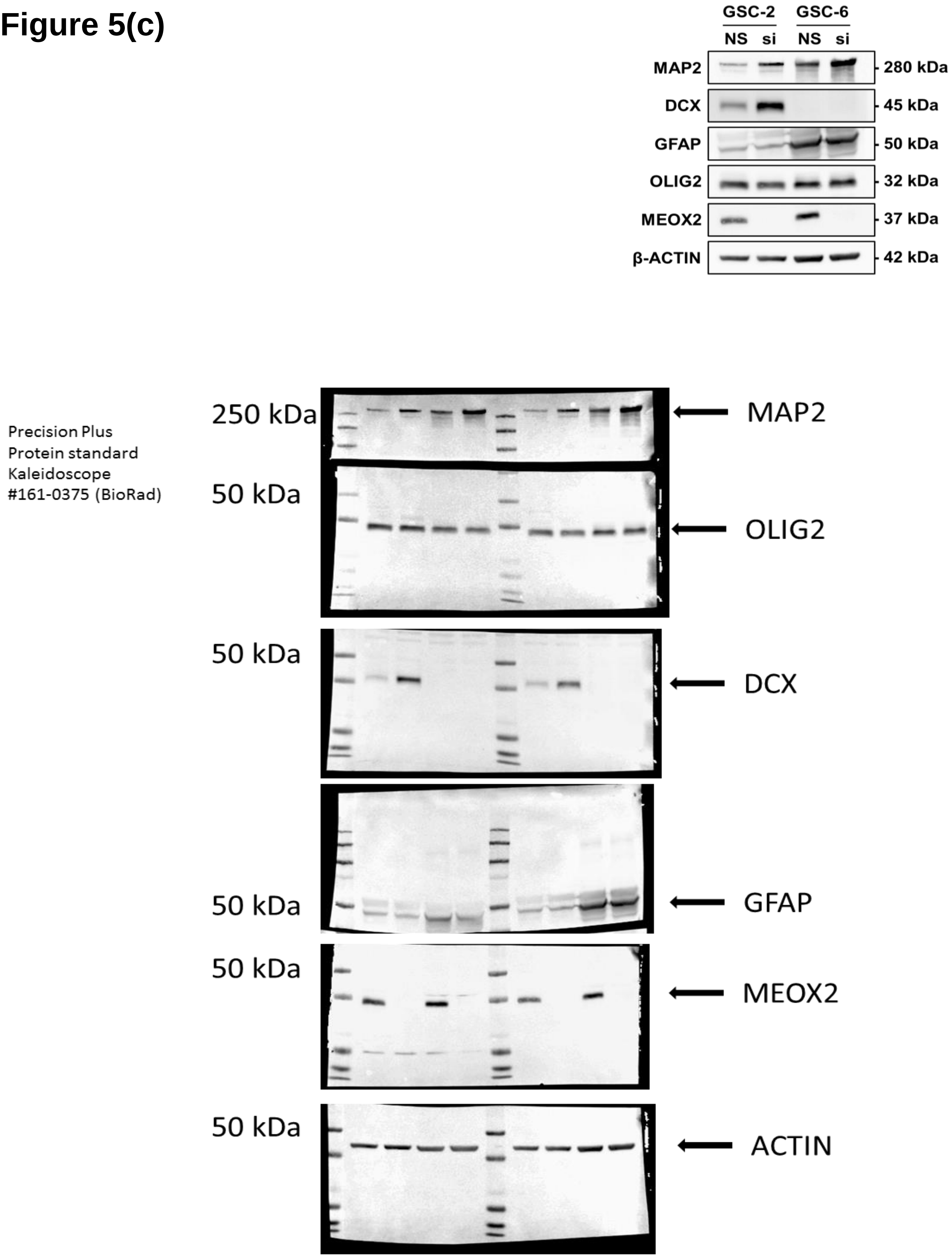
GSC-2
GSC-6
NS
si
NS
si
MAP2
DCX
GFAP
OLIG2
MEOX2
β-ACTIN
280 kDa
45 kDa
50 kDa
32 kDa
37 kDa
42 kDa
Precision Plus
Protein standard
Kaleidoscope
#161-0375 (BioRad)
250 kDa
50 kDa
50 kDa
50 kDa
50 kDa
50 kDa
MAP2
OLIG2
DCX
GFAP
MEOX2
ACTIN

Figure 6(a)

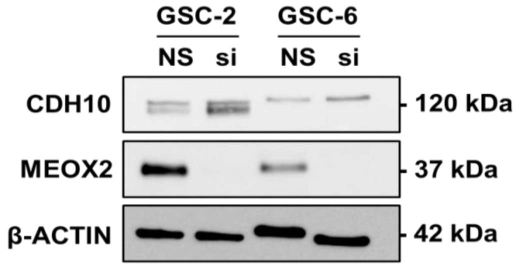

Precision Plus
Protein standard
Kaleidoscope
#161-0375 (BioRad)

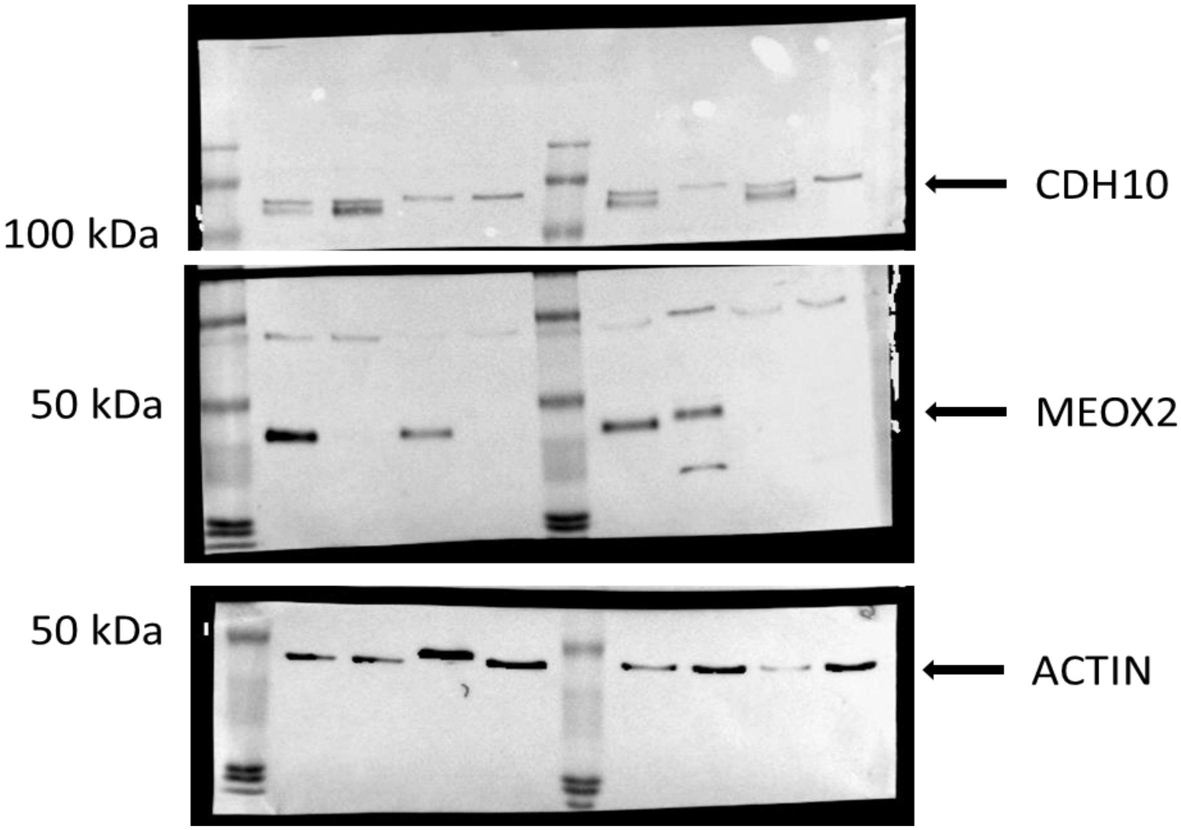

Figure 6(c)

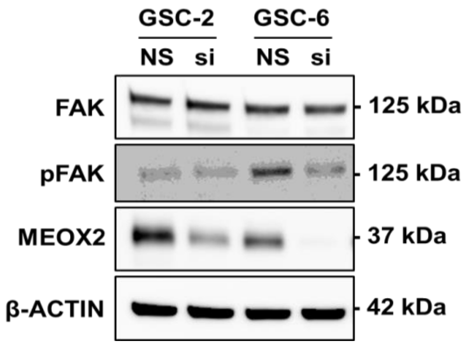
GSC-2
GSC-6
NS
si
NS
si
FAK
pFAK
MEOX2
β-ACTIN
125 kDa
125 kDa
37 kDa
42 kDa

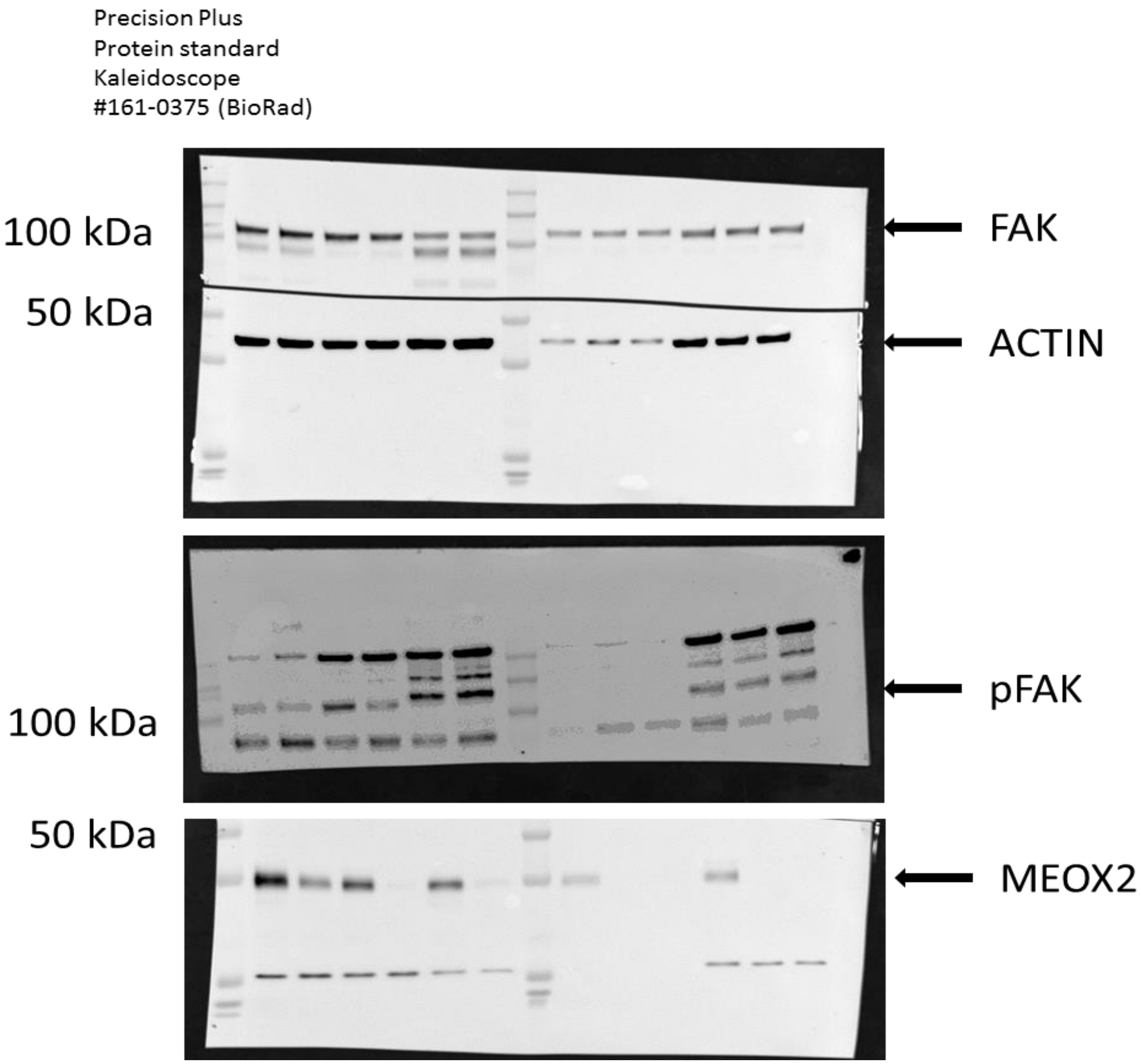
Precision Plus
Protein standard
Kaleidoscope
#161-0375 (BioRad)
100 kDa
50 kDa
FAK
ACTIN
pFAK
100 kDa
50 kDa
MEOX2

Figure S1

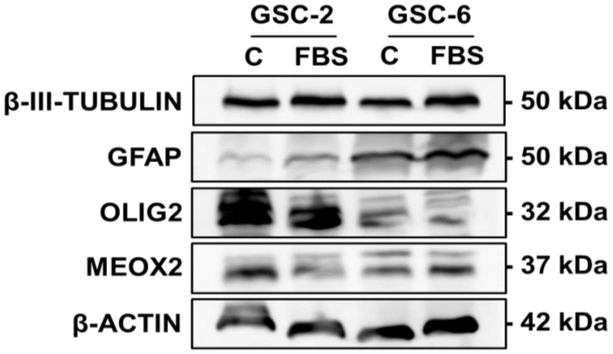

Precision Plus
Protein standard
Kaleidoscope
#161-0375 (BioRad)

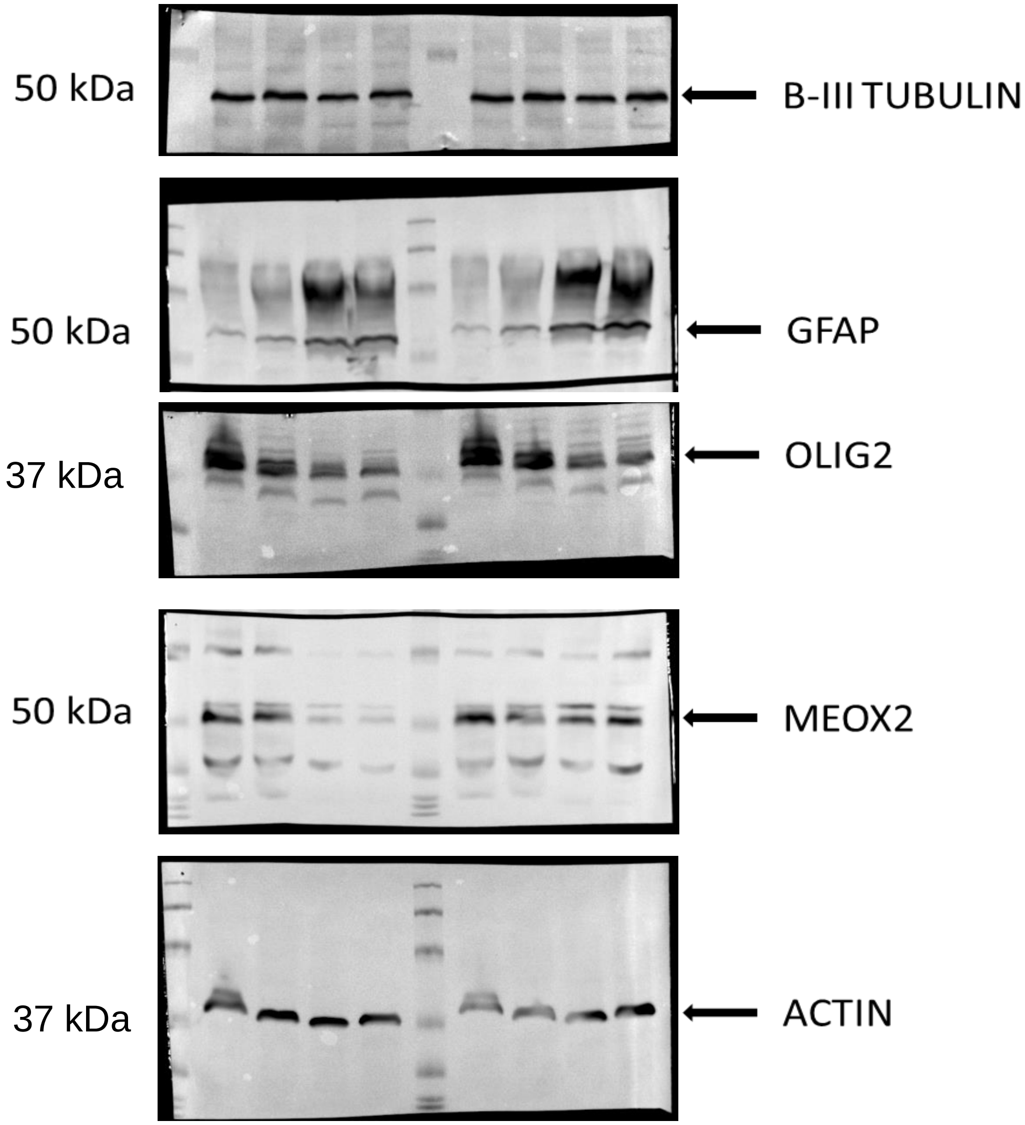

Supplement: Supplementary file 1 [file cancers-13-05943-s001.zip › cancers-1387661-supplementary.pdf]
